# Comparative pharmacokinetics of porcine and human anti-influenza hemagglutinin monoclonal antibodies in outbred pigs and minipigs

**DOI:** 10.3389/fimmu.2024.1471412

**Published:** 2024-10-31

**Authors:** Basudev Paudyal, Elliot Moorhouse, Bhawna Sharma, Michael Dodds, Victor Nguyen, Mark Milad, Elma Tchilian

**Affiliations:** ^1^ Host Responses, The Pirbright Institute, Woking, United Kingdom; ^2^ Integrated Drug Development, Certara, Radnor, PA, United States; ^3^ Milad Pharmaceutical Consulting LLC, Plymouth, MI, United States

**Keywords:** pharmacokinetic, anti-influenza monoclonal antibodies, minipigs, outbred pigs, 2-12C, pb27

## Abstract

Assessing the pharmacokinetics of monoclonal antibodies (mAbs) in relevant animal models is essential for designing improved formulations and developing mAb delivery platforms. We have established the pig, a large natural host animal for influenza with many similarities to humans, as a robust model for testing the therapeutic efficacy of anti-influenza mAbs and evaluating mAb delivery platforms. Here, we compared the pharmacokinetic characteristics of two anti-influenza hemagglutinin mAbs, human 2-12C and porcine pb27, in Göttingen minipigs and Landrace × Large White outbred pigs. Minipigs offer the advantage of a more stable weight, whereas outbred pigs are more readily available but exhibit rapid growth. Outbred pigs and minipigs showed similar pharmacokinetics and a similar porcine pb27 half-life (half-life of 15.7 days for outbred pigs and 16.6 days for minipigs). In contrast, the half-life of human 2-12C was more rapid in two of the minipigs but not in the outbred pigs, correlating with the development of antidrug antibodies in the two minipigs. Our results demonstrate that both outbred pigs and minipigs are appropriate models for pharmacokinetic studies and the evaluation of mAb delivery platforms, potentially bridging the gap between small animals and human trials.

## 1 Introduction

Monoclonal antibodies (mAbs) are highly effective therapeutics for a wide range of diseases and often used to complement immunization in the treatment of infections. The pharmacokinetic (PK) characteristics of therapeutic mAb are crucial for designing improved formulations. Rodents and non-human primates are generally the species of choice when characterizing mAb PK, despite known immunological and physiological differences between humans and these animals (1–5). However, pigs offer a potentially better predictive animal model due to their many physiological, immunological, genetic, and anatomical similarities to humans, making them a valuable biomedical tool for toxicology, PK, dermatology, cardiovascular system pulmonary infections, and organ transplantation (6–15).

Göttingen minipigs have been used extensively to predict PK parameters and drug safety in humans (16–20), and recently, a humanized minipig model has been established to circumvent possible limitations in evaluating therapeutic recombinant human mAbs (21). However, outbred pigs, which are more readily available and cheaper, differ mainly from minipigs in their growth rates and size at sexual maturity with no major differences in the immune system, making them an appropriate model for testing mAbs and mAb delivery platforms (20, 22). However, to date, only one study in outbred pigs to assess the PKs of a subcutaneously administered mAb (ustekinumab) has been published (22). We have used Landrace × Large White outbred pigs to establish a robust influenza model to evaluate the therapeutic potential and delivery platforms for mAbs (23) and generated the first porcine influenza-specific anti-hemagglutinin (HA) mAbs, showing that the outbred pigs recognize the same epitopes as human mAbs (24).

Using outbred pigs, we demonstrated the protective efficacy of the strongly neutralizing pH1N109 (pH1N1) specific anti-hemagglutinin mAbs human 2-12C and porcine pb27 in the pig influenza challenge model. Both mAbs bind to the K130E hemagglutinin site of pH1N1. Prophylactic intravenous administration of recombinant 2-12C or pb27 significantly reduced nasal shedding, the lung viral load, and lung pathology following a pH1N1 influenza challenge (23–25). Therefore, both human 2-12C and porcine pb27 mAbs are excellent tools to evaluate mAb delivery platforms in this large natural host animal model for influenza. However, previous studies were usually terminated at 5 days post mAb administration and the long-term potency and PK properties of these mAbs were not analyzed.

Although the PK properties of mAbs or biologics have been extensively investigated in minipigs, a direct comparison with outbred pigs has not been performed. Here, we evaluated head-to-head the PKs of human 2-12C and porcine pb27 mAbs in Landrace × Large White outbred pigs and Göttingen minipigs to determine whether outbred pigs, despite their rapid growth, can serve as a practical and more accessible alternative to the more expensive and less readily available minipigs.

## 2 Materials and methods

### 2.1 Antibody preparation

The anti-influenza human IgG1 mAb 2–12C and porcine IgG1 mAb pb27 were produced in bulk by Absolute Ab Ltd (Redcar, UK). They were dissolved in 25 mM histidine, 150 mM NaCl, and 0.02% Tween P80 (pH 6) diluent.

### 2.2 Animal studies

All experiments were approved by the ethical review processes at the Pirbright Institute and Reading University and conducted according to the UK Government Animal (Scientific Procedures) Act 1986 supported by Project License P47CE0FF2. Eight 6-week-old Landrace × Large White female pigs were obtained from a commercial high-health status herd, which is regularly screened for the absence of influenza A infection by hemagglutination inhibition using four swine influenza virus antigens from H1N1pdm09, H1N2, H3N2, and avian-like H1N1. Eight 18-week-old high-health status influenza-free female minipigs were obtained from Ellegaard Göttingen Minipigs, Denmark. Landrace × Large White pigs are referred to as “outbred” and the Ellegaard Göttingen Minipigs as “minipigs” throughout the text.

**Figure 1:**
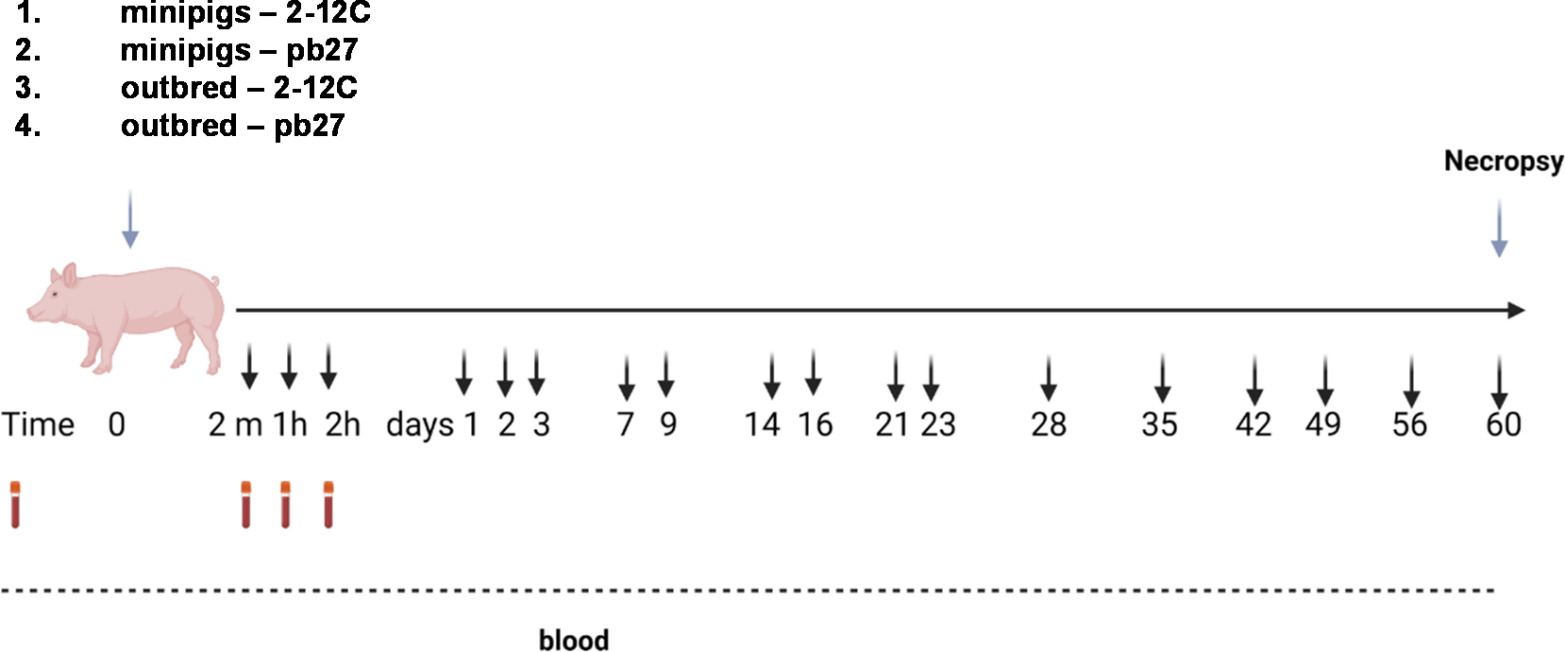
Experimental design. Recombinant 2-12C or pb27 at 3.5 mg/kg were administered intravenously to four minipigs or four outbred pigs. Clotted blood was obtained at the indicated times.

Outbred pigs were acclimatized for 14 days and minipigs for 18 days. The outbred and minipigs were randomized into two groups of four pigs each. A group of minipigs and a group of outbred pigs were given 3.5 mg/kg 2-12C mAb by intravenous bolus (IV). The remaining groups of minipigs and outbred pigs were given 3.5 mg/kg pb27 mAb I.V. The rec 2-12C and pb27 were administered to the ear vein following sedation with 2 mg/kg Zoletil (zolazepam) and 4 mg/kg Stresnil (azaperone). The animals were bled at 2 min, 1 h, and 2 h post administration, daily for the first 3 days followed by twice per week for 3 weeks and weekly for the last 5 weeks (**Figure 1**). The pigs were not sedated for bleeding except on the first day. The pigs’ weights were recorded each day blood was collected. The study was terminated at day 60 post administration.

### 2.3 ELISA

**Figure 2:**
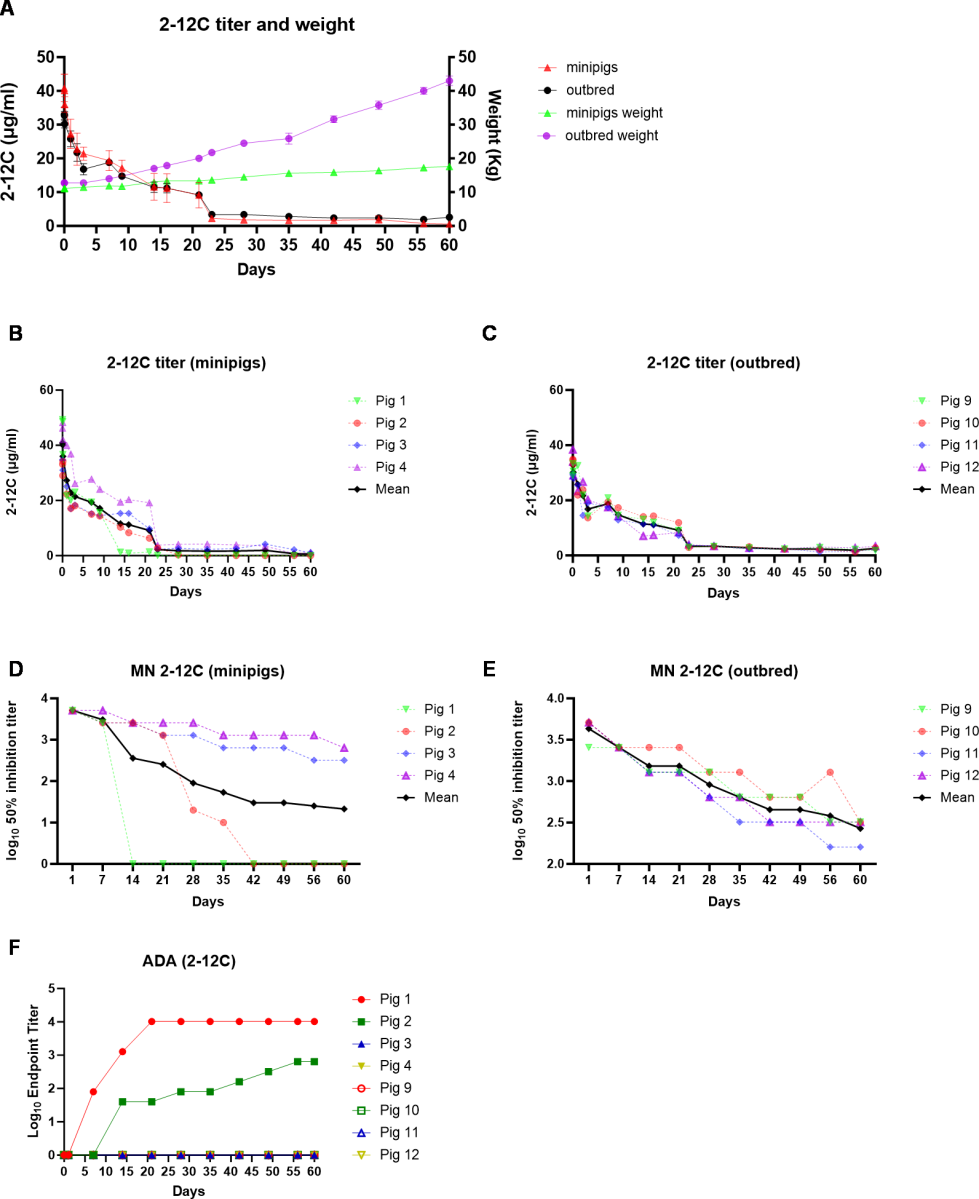
PK of 2-12C in outbred and minipigs. Overall representation of weight and 2-1C concentration in outbred and minipigs **(A)**. 2-12C serum ELISA titers in individual minipigs **(B)** and outbred pigs **(C)**. Neutralizing titers in the serum of 2-12C treated pigs. Fifty percent inhibition titers of individual minipigs **(D)** and outbred pigs **(E)** over the time course. ADA responses in both outbred and minipigs **(F)**.

The concentration of 2-12C and pb27 was evaluated in serum using an ELISA against recombinant HA protein of A/Eng/195/2009 hemagglutinin (**Figure 2**). Briefly, 100 µl of 1 µg/ml recombinant HA protein was coated on ELISA plates overnight at 4°C. Plates were washed with 0.05% Tween 20 in PBS (PBS-T) and blocked with 4% semi-skimmed milk for 2 h. The serum samples were diluted in 4% semi-skimmed in PBS-T, and 100 µl of diluted serum sample was added to the wells for 1 h on a rocking platform at room temperature (RT). A standard curve of rec 2-12C or pb27 was prepared as 1:2 serial dilutions starting at 1 µg/ml in 4% semi-skimmed milk and added in duplicate. Plates were washed and incubated with goat anti-human IgG-Fc Ab HRP (Bethyl laboratories, A80-104P) or goat anti-pig IgG-Fc-HRP (Bio-Rad, AA141) for 1 h with shaking at room temperature. Plates were washed again and developed with 50 µl TMB substrate at RT. The reaction was stopped with 50µl 1M H_2_SO_4_. The ODs were read at 450 nm and 630 nm (reference wavelength) using a Biotek Elx808 reader. The serum concentration was interpolated from the standard curve using a sigmoidal four-parameter logistic curve fit for the log of the concentration.

### 2.4 Microneutralization

The functional activity of 2-12C and pb27 in serum was measured using a microneutralization (MN) assay. Serum samples were heat-treated for 30 min at 56°C. Fifty microliters of serially diluted samples were incubated with an equal volume of pH1N1 in a 96-well flat tissue culture plate. The virus was titrated beforehand in the absence of serum to determine the PFU/ml necessary to yield a plateau infection in the MN assay. After 2 h, 100 µl of MDCK SIAT-1 cells at 3×10^5^/ml cells per well was added and incubated for 18 h. Cells were fixed and permeabilized and stained with anti-NP (clone: AA5H, Bio-Rad Laboratories) followed by goat anti-mouse IgG HRP (P0447, Dako) Ab. After the addition of the TMB substrate, the reaction was stopped with 1 M H_2_SO_4_, and absorbance was measured at 450 and 630 nm (reference wavelength) using a Biotek Elx808 reader. The MN titers were expressed as the half maximal inhibitory dilution (50% inhibitory titer is the midpoint between uninfected control wells and virus-infected positive controls) derived by linear interpolation from neighboring points in the titration curve.

### 2.5 The anti-drug antibody response

The ADA against 2-12C was measured using an ELISA-based assay. ELISA plates wells were coated with 100 µl of 1 µg/ml of HA overnight at 4**°**C. Plates were washed with PBS-T and blocked with 4% semi-skimmed milk PBS-T for 1 h. One hundred microliters of 1 µg/ml of 2-12C was added to the wells and incubated for 1 h at RT. The plates were washed again. Serially diluted serum samples were added to the wells and incubated for 1 h on a rocking platform at RT. The plates were washed again with PBS-T and incubated with mouse anti-pig IgG HRP (Mabtech) for 1 h. The wells were washed and developed using TMB substrate at RT. The reaction was stopped with 1M H_2_SO_4_. The ODs were read at 450 nm and 630 nm (reference wavelength) using a Biotek Elx808 reader. Samples were measured as the end point titer representing the highest dilution with a signal greater than the cutoff. The cutoff value was defined as twice the average OD of pre-bleed serum samples plus the standard deviation.

### 2.6 PK data analysis

The data for serum mAb concentration over time were analyzed by non-compartmental PK analysis performed in Phoenix WinNonlin (version 8.4.0.1672, Certara L.P., Princeton, NJ, USA). The AUC was calculated using the linear-up log-down calculation method for intravenous bolus injection. The following parameters were measured or estimated: peak concentration following dose (C_max_), area-under-the-curve extrapolated to infinity (AUC_inf_), half-life (T_1/2_), and clearance (CL). Statistical analyses were limited to descriptive statistics such as mean and standard deviation, if applicable. T_1/2_ and AUC_inf_ was only estimated when R^2^adj ≥0.8, span ≥2, and %AUCext ≤20. The total dose in milligrams was determined using the starting body weight of each pig. The dose-normalized peak concentration (C_max_/D) and area-under-the-curve to infinity (AUC_inf_/D) were calculated using the total dose.

## 3 Results

### 3.1 The pharmacokinetics of human 2-12C mAb in outbred and minipigs

Recombinant 2-12C was administered intravenously at 3.5 mg/kg to groups of four minipigs (average weight 11.12 kg) and four outbred pigs (average weight 12.75 kg). Serum PK samples were collected at 2 min, 1 and 2 h post administration, daily for the first 3 days followed by twice per week for 3 weeks and weekly for the last 5 weeks (**Figure 1**). The weight of the minipigs increased from 11.12 kg to 17.6 kg (58.3%) and the outbred pigs from 12.75 kg to 43 kg (237%) (**Figure 2A**). The mean serum titers for 2-12C or pb27 over time were evaluated by an ELISA (**Figure 2**). The PK parameters were estimated by non-compartmental analysis (**Table 1**, **Supplementary Table 1**). The maximal concentration at 2 min post administration was 33.9 µg/ml for outbred pigs and 41.0 µg/ml for minipigs (**Table 1**). The half-life (T_1/2_) of 2-12C was rapid and serum concentrations decreased to a concentration that was less than measurable in two out of four minipigs on days 14 and 42. The remaining two minipigs and all outbred pigs showed a gradual decline in serum 2-12C concentration to 2.1 µg/ml at day 60 (**Figures 2B, C**). The functionality of 2-12C in sera samples was determined by a microneutralization assay. Minipigs 1 and 2 did not show neutralizing activity after days 14 and 42, respectively, in line with the ELISA data (**Figure 2D**). In the remaining animals, the neutralizing activity decreased over time in a similar manner to 2-12C ELISA titers, showing average 50% inhibition titers of 1:346 at day 60 post administration (**Figures 2D, E**). A bridging ELISA was performed to assess the anti-drug antibody (ADA) response. Minipigs 1 and 2 developed an ADA response, whereas the other minipigs and outbred pigs did not (**Figure 2F**).

**Table 1:** Geometric mean (CV%) parameters for 2-12C in outbred and minipigs.

PK parameters	2-12C minipig	2-12C outbred
Dose (mg/kg)	3.5	3.5
N	4	4
C_max_, µg/ml	41.0 (20.3)	33.9 (10.8)
C_max_/Dose, µg/ml/mg	1.05 (21.7)	0.764 (18.6)
T_max_, hr, median (range)	0.517 (0.0333, 2.00)	0.0333 (0.0333, 1.00)
AUC_inf_, day*µg/ml	356 (61.6)	473 (7.59)
AUC_inf_/Dose, day*µg/ml/mg	9.25 (61.6)	10.2 (7.06)
T_1/2_, day	5.72 (64.7)	14.7 (1.94)

The geometric mean terminal T_1/2_ of 2-12C was 14.71 days in outbred pigs and 5.72 days in minipigs. The two minipigs that developed ADA had a T_1/2_ that was much shorter than the one remaining minipig (**Supplementary Table 1**). In contrast, mAb T_1/2_ was more consistent in all outbred pigs with a geometric mean T_1/2_ of 14.7 days. Minipigs, with ADA present in the group, had highly variable clearance (CL) that ranged from 0.06 to 0.17 L/day, whereas outbred pigs were consistent among each other, ranging from 0.09 to 0.11 L/day (**Supplementary Table 1**). The area under the concentration time curve AUC_inf_ was similar between minipigs and outbred pigs at 356 and 473 day*µg/ml, respectively. ADA did not affect peak concentrations but increased CL, resulting in a lower systemic exposure as measured by AUC_inf_ than the one minipig without ADA and the outbred pigs.

### 3.2 The pharmacokinetics of porcine pb27 mAb in outbred and minipigs

**Figure 3:**
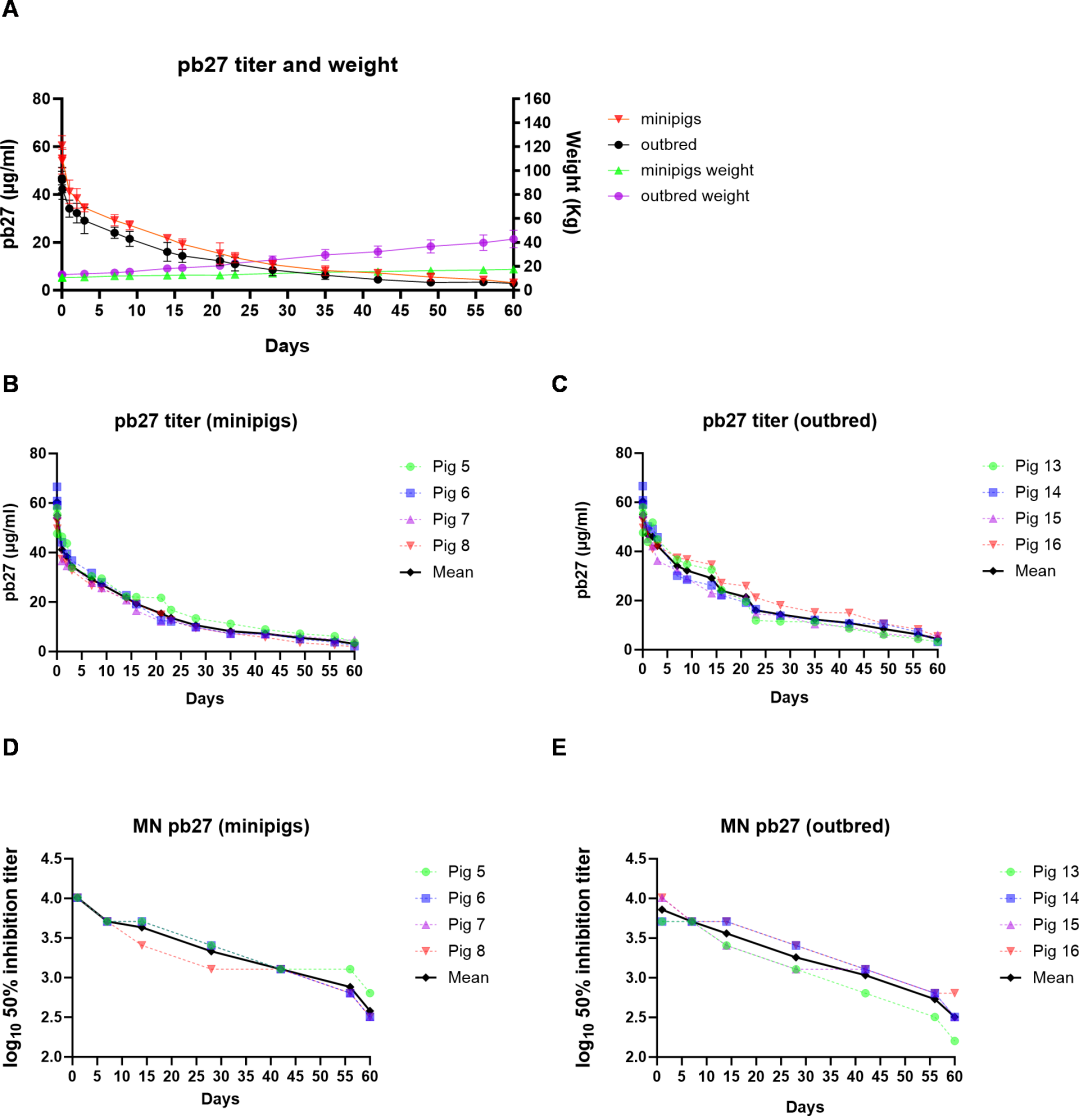
PK of pb27 in outbred and minipigs. Overall representation of weight and pb27 concentration in outbred and minipigs **(A)**. pb27 serum ELISA titers in individual minipigs **(B)** and outbred pigs **(C)**. Neutralizing activity in the serum of pb27-treated pigs. Fifty percent inhibition titers of individual minipigs **(D)** and outbred pigs **(E)** over the time course.

The PKs of porcine pb27 were examined in outbred and minipigs in a similar fashion to 2-12C, following a 3.5 mg/kg intravenous administration (**Figure 1**). The weight of the minipigs increased from 10.62 kg to 17.5 kg (64.8%), and the weight of the outbred pigs increased from 13 to 42.75 kg at day 60 (229%) (**Figure 3A**). The PK parameters were estimated by non-compartmental analysis (**Table 2**, **Supplementary Table 2**). A peak pb27 concentration of 60.4 µg/ml was observed in minipigs and 48.8 µg/ml in outbred pigs at 2 min after administration, which declined to 3.1 µg/ml in minipigs and 2.8 µg/ml in outbred pigs at day 60 (**Figures 3B, C**). Neutralizing activity decreased over time in a similar manner to pb27 ELISA titers. The peak average 50% inhibition neutralization titer at day 1 was 1:8,610 which declined to 1:349 at day 60 post administration (**Figures 3D, E**). The terminal T_1/2_ was more than 2 weeks in both pig breeds. The T_1/2_ values of pb27 in minipig (16.6 days) were comparable with outbred (15.7 days) (**Table 2**). The AUC_inf_ showed a greater systemic exposure in minipigs (931 day*µg/ml) than outbred (726 day*µg/ml). Similarly, CL differed in minipigs and outbred pigs with an average of 0.040 and 0.063 L/day, respectively. The lack of ADA following pb27 administration shows in the consistency of PK parameters when considering weight for minipigs and outbred pigs. The larger species had lower serum concentrations and higher clearance.

**Table 2:** Geometric mean (CV%) parameters for pb27 in outbred and minipigs.

PK parameters	pb27 minipig	pb27 outbred
Dose (mg/kg)	3.5	3.5
N	4	4
C_max_, µg/ml	60.4 (6.54)	48.8 (5.93)
C_max_/Dose, µg/ml/mg	1.64 (21.58)	1.07 (7.39)
T_max_, hr, median (range)	0.0333 (0.0333, 0.0333)	0.0333 (0.0333, 1.00)
AUC_inf_, day*µg/ml	931 (12.5)	726 (16.0)
AUC_inf_/dose, day*µg/mL/mg	25.2 (20.5)	16.0 (21.2)
T_1/2_, day	16.6 (14.9)	15.7 (17.7)

Dose (mg/kg), amount of the drug administered to each pig in milligrams per kilogram of body weight; T_max_ (day), time to reach the maximum concentration (Cmax) in the bloodstream after administration; C_max_ (ug/ml), maximum concentration of the drug in the bloodstream after administration; C_max_/D, ratio of Cmax to the total administered dose; AUC_inf_ (day*ug/ml), total drug exposure from time of drug administration to the end of the study and estimated to the time the drug is eliminated from the body; AUC_inf_/D, ratio of AUCinf to the total administered dose; T_1/2_ (day), terminal half-life of the drug; CV%, the coefficient of variation percentage, also known as the normalized root-mean-square deviation, describes the extent of variability in relation to the mean.

## 4 Discussion

We performed a comparative PK study of human and porcine anti-influenza mAbs in Landrace × Large White outbred and Göttingen minipigs and observed similar pharmacokinetics and T_1/2_ of the porcine pb27 mAb in both breeds. In contrast, human 2-12C mAb had a shorter T_1/2_ in two out of four minipigs, but not in outbred pigs, correlating with the presence of ADA in the two minipigs with higher clearance. 2-12C was detectable at 60 days in the remaining minipigs and all outbred pigs.

ADA is a complex immune process that involves antigen recognition, T- and B-cell activation, antibody production, and effector functions that can impact the efficacy and safety of the therapeutic mAb. ADA can accelerate the clearance of the therapeutic antibody from the system, reducing its efficacy by decreasing its half-life in circulation. The lack of ADA in two minipigs and four outbred pigs may indicate that ADA is a low frequency event and/or genetic or age differences. Additionally, the intravenous route of administration may partially circumvent the antigen recognition and development of an immune response as subcutaneous, but not intravenous, administration of therapeutic human IgG1 against TNF-alpha (adalimumab) to minipigs resulted in an ADA response (26). In the same study, eight other therapeutic human antibodies against unspecified targets did not induce ADA after either intravenous or subcutaneous administration. Another study indicated that ADA induction following the subcutaneous administration of adalimumab to minipigs is dose dependent (1). In contrast, a single intravenous administration of humanized therapeutic anti-CD49d (natalizumab) against minipigs induced ADA, irrespective of the dose (27). However, there are still major gaps in the understanding of the pharmacokinetics of ADAs, their neutralizing ability, and cross-reactivity with endogenous molecules.

In addition, differences in environmental controls between Göttingen minipigs and Landrace × Large White outbred pigs may also be contributing factors for the observed differences in ADA. Göttingen minipigs are raised in a highly controlled environment with stringent biosecurity measures, which significantly reduces their exposure to pathogens and environmental antigens. In contrast, outbred pigs often experience more variable and less controlled conditions, which can lead to differences in immune system priming and maturation. Although the minipigs were acclimatized for 18 days under the same conditions as the outbred pigs, it might be expected that the limited exposure of minipigs to environmental antigens would make them less likely to respond to the mAbs. The fact that minipigs produced ADA suggests that genetic factors or the age of the minipigs versus the outbred pigs (20 weeks versus 8 weeks) are important. Outbred pigs were not immunologically mature at 8 weeks of age. Future studies using a larger number of age-matched animals, SLA typing, an understanding of TCR and BCR diversity, cytokine production, and immune-regulatory gene expression would be required to understand the genetic predisposition of different pig breeds to help select the appropriate animal model.

The administration of human anti-influenza MHAA4549A mAb intravenously to cynomolgus monkeys did not induce an ADA response, possibly due to lack of endogenous host targets, and the serum T_1/2_ was 11.9 days at a low dose of 5 mg/kg and 11.3 days at a high dose of 100 mg/kg (28). Circulating IgG is protected from systemic clearance by FcRn receptors, which prevent lysosomal degradation within cells (29), and it has been shown that the binding affinity of pig, cynomolgus monkey, and human FcRn receptors to human IgGs are very similar (26). Given this similarity, pharmacokinetic parameters scale relative to the weight of the species. For mAb with a 21-day T_1/2_ in humans (70 kg), we would expect a T_1/2_ of 11, 14, and 17 days in cynomolgus monkeys (5 kg), minipigs (15 kg), and outbred pigs (30 kg), respectively. This could be one of the reasons for the similar T_1/2_ of anti-influenza mAb observed in cynomolgus monkeys (11.3 days) (28) and outbred pigs (13.1–19.7) in our study, providing an additional rationale for the use of outbred pigs as a translatable species for mAb PK studies, as observed parameters in heavier mammals are closer to those of humans. Adalimumab has a T_1/2_ in humans of approximately 10–20 days, found across several studies (30, 31). When scaled on body weight to minipigs and outbred pigs, we would estimate T_1/2_ values of 6.8–13.6 and 8.1–16.2 days, respectively, comparable with the observed T_1/2_ of pb27 in our study (15.7–16.6 days).

In the present study, we used mAbs against influenza virus hemagglutinin, which is not an in-target antigen in humans or pigs. However, mAbs can be used prophylactically to protect against infection, and an important issue is how long effective prophylactic concentrations could be maintained. The use of out-of-target mAbs as in this study allows the baseline PK to be established and non-specific interactions between the infused mAb and immune system to be identified. Furthermore, it is well-known that target-mediated drug disposition (TMDD) plays a role in the PK of antibodies targeting cell-membrane antigens. Many factors impact the degree of TMDD: the abundance of the target, the degree to which the antibody can access the biological space of the target, the kinetics of the unbound target on the cell surface (i.e., receptor turnover), and the kinetics of the bound target on the cell surface (i.e., receptor internalization, receptor shedding). As such, it is extremely difficult to predict with reasonable accuracy the impact TMDD will have (32, 33). Generally, the accuracy of these efforts is poor, unless the dose level is sufficiently high as to saturate the non-linear elimination pathway such that the linear clearance process is dominant. Under these saturating conditions, these antibodies have PKs that behave much like an antibody directed at soluble targets.

Our study examines Landrace × Large White outbred and Gottingen minipigs for their application in translational PK studies. As stated above, antibodies directed at cell-membrane targets exhibit increased clearance (elimination) relative to antibodies against soluble targets. However, at sufficiently high doses, cell-membrane targets can be swamped, and clearance becomes similar to antibodies directed against soluble targets. As such, we can think of PK predictions in outbred pigs or minipigs as a *lower bound* on the human clearance. That is, at low doses in the clinic, the drug will be cleared more rapidly than expected from the, for example, minipig data. It may not be possible to predict what “more rapidly” means exactly, but this same problem is evident in all other preclinical species. However, as the clinical dose level increases, the clearance will decrease and asymptotically approach the preclinical prediction. Antibodies are often dosed such that the cell-membrane target is saturated (with the aim of producing a complete pharmacological response), and so we recover a clearance estimate that is somewhat larger than predicted from the preclinical model in which cell-membrane binding is not present.

In summary, our results confirm that both outbred pigs and minipigs are appropriate models for PK studies of porcine or human mAbs. Minipigs offer the advantage of a more stable weight but are significantly more expensive. In contrast, outbred pigs are readily available at low cost and increase in body weight rapidly, but this does not appear to affect the rate of decline in antibody titers. We also demonstrate that the human 2-12C and porcine pb27 mAbs are very useful tools for evaluating mAb delivery platforms in large animals. Using pigs as models can reduce the reliance on non-human primates, addressing the ethical concerns and practical challenges of primate research, and bridge the gap between preclinical studies in small animals and human clinical trials.

## Data Availability

The original contributions presented in the study are included in the article/**Supplementary Material**. Further inquiries can be directed to the corresponding authors.
