# Supplementary material for: Comparative pharmacokinetics of porcine and human anti-influenza hemagglutinin monoclonal antibodies in outbred pigs and minipigs
Source: Front Immunol. 2024 Oct 31;15:1471412. doi: 10.3389/fimmu.2024.1471412 (PMC11560753; doi:10.3389/fimmu.2024.1471412)
Supplement: Supplementary file 1 [file Table1.docx]

**Supplementary Table 1**

**PK parameters of 2-12C in outbred and minipigs**

| **Pig Type** | **Drug** | | **Pig** | **Dose**  **(mg)** | **T_max_**  **(hr)** | **C_max_**  **(ug/mL)** | **C_max_/D**  **(ug/mL/mg)** | **AUC_inf_**  **(day*ug/mL)** | **AUC_inf_/D**  **(day*ug/mL/mg)** | **T_1/2_**  **(day)** | **V_d_**  **(L)** | **CL**  **(L/day)** |
| --- | --- | --- | --- | --- | --- | --- | --- | --- | --- | --- | --- | --- |
| Minipig | 2-12C | | 1 | 38.50 | 0.03 | 49.29 | 1.28 | 229.02 | 5.95 | 3.88 | 0.94 | 0.17 |
|  | | 2 | | 38.50 | 2.00 | 34.18 | 0.89 | 292.53 | 7.60 | 4.28 | 0.81 | 0.13 |
|  |  | 3 | | 40.25 | 0.03 | 34.73 | 0.86 |  |  |  |  |  |
|  |  | 4 | | 38.50 | 1.00 | 48.28 | 1.25 | 675.11 | 17.54 | 11.30 | 0.93 | 0.06 |
|  |  | N | | 4.00 | 4.00 | 4.00 | 4.00 | 3.00 | 3.00 | 3.00 | 3.00 | 3.00 |
|  |  | Min | | 38.5 | 0.0333 | 34.2 | 0.863 | 229 | 5.95 | 3.88 | 0.812 | 0.0570 |
|  |  | Median | | 38.5 | 0.517 | 41.5 | 1.07 | 293 | 7.60 | 4.28 | 0.930 | 0.132 |
|  |  | Max | | 40.3 | 2.00 | 49.3 | 1.28 | 675 | 17.5 | 11.3 | 0.940 | 0.168 |
|  |  | Geometric Mean | | 38.93 | 0.217 | 41.00 | 1.053 | 356.3 | 9.254 | 5.724 | 0.8923 | 0.1081 |
|  |  | Geometric CV% | | 2.223 | 1077 | 20.31 | 21.65 | 61.56 | 61.56 | 64.69 | 8.147 | 61.56 |

| **Pig Type** | **Drug** | **Pig** | **Dose**  **(mg)** | **T_max_**  **(hr)** | **C_max_**  **(ug/mL)** | **C_max_/D**  **(ug/mL/mg)** | **AUC_inf_**  **(day*ug/mL)** | **AUC_inf_/D**  **(day*ug/mL/mg)** | **T_1/2_**  **(day)** | **V_d_**  **(L)** | **CL**  **(L/day)** |
| --- | --- | --- | --- | --- | --- | --- | --- | --- | --- | --- | --- |
| Outbred | 2-12C | 9 | 50.75 | 1.00 | 33.29 | 0.66 | 476.84 | 9.40 | 14.99 | 2.30 | 0.11 |
|  | | 10 | 47.25 | 0.03 | 34.89 | 0.74 | 508.29 | 10.76 | 14.73 | 1.98 | 0.09 |
|  |  | 11 | 42.00 | 0.03 | 29.64 | 0.71 | 437.06 | 10.41 | 14.43 | 2.00 | 0.10 |
|  |  | 12 | 38.50 | 0.03 | 38.38 | 1.00 |  |  |  |  |  |
|  |  | N | 4.00 | 4.00 | 4.00 | 4.00 | 3.00 | 3.00 | 3.00 | 3.00 | 3.00 |
|  |  | Min | 38.5 | 0.0333 | 29.6 | 0.656 | 437 | 9.40 | 14.4 | 1.98 | 0.0930 |
|  |  | Median | 44.6 | 0.0333 | 34.1 | 0.722 | 477 | 10.4 | 14.7 | 2.00 | 0.0961 |
|  |  | Max | 50.8 | 1.00 | 38.4 | 0.997 | 508 | 10.8 | 15.0 | 2.30 | 0.106 |
|  |  | Geometric Mean | 44.37 | 0.07801 | 33.90 | 0.7640 | 473.2 | 10.17 | 14.71 | 2.087 | 0.09833 |
|  |  | Geometric CV% | 12.31 | 412.7 | 10.77 | 18.55 | 7.590 | 7.062 | 1.937 | 8.525 | 7.062 |

*PK parameters for pig 3 (minipig) and pig 12 (outbred) could not be reliably estimated due to failure to meet the criteria of R2adj ≥0.8, span ≥2, and %AUCext ≤20 as set in PK data analysis criteria.*

**Supplementary Table 2**

**PK parameters of pb27 in outbred and minipigs following**

| **Pig Type** | **Drug** | **Pig** | **Dose**  **(mg)** | **T_max_**  **(hr)** | **C_max_**  **(ug/mL)** | **C_max_/D**  **(ug/mL/mg)** | **AUC_inf_**  **(day*ug/mL)** | **AUC_inf_/D**  **(day*ug/mL/mg)** | **T_1/2_**  **(day)** | **V_d_**  **(L)** | **CL**  **(L/day)** |
| --- | --- | --- | --- | --- | --- | --- | --- | --- | --- | --- | --- |
| Minipig | PB27 | 5 | 36.75 | 0.03 | 57.95 | 1.58 | 1095.47 | 29.81 | 18.51 | 0.90 | 0.03 |
|  | | 6 | 29.75 | 0.03 | 66.56 | 2.24 | 896.07 | 30.12 | 15.03 | 0.72 | 0.03 |
|  |  | 7 | 42.00 | 0.03 | 59.13 | 1.41 | 938.79 | 22.35 | 19.07 | 1.23 | 0.04 |
|  |  | 8 | 40.25 | 0.03 | 58.35 | 1.45 | 813.43 | 20.21 | 14.17 | 1.01 | 0.05 |
|  |  | N | 4.00 | 4.00 | 4.00 | 4.00 | 4.00 | 4.00 | 4.00 | 4.00 | 4.00 |
|  |  | Min | 29.8 | 0.0333 | 57.9 | 1.41 | 813 | 20.2 | 14.2 | 0.720 | 0.0332 |
|  |  | Median | 38.5 | 0.0333 | 58.7 | 1.51 | 917 | 26.1 | 16.8 | 0.954 | 0.0391 |
|  |  | Max | 42.0 | 0.0333 | 66.6 | 2.24 | 1100 | 30.1 | 19.1 | 1.23 | 0.0495 |
|  |  | Geometric Mean | 36.87 | 0.03333 | 60.40 | 1.638 | 930.5 | 25.24 | 16.56 | 0.9465 | 0.03963 |
|  |  | Geometric CV% | 15.44 | 0.000 | 6.538 | 21.58 | 12.46 | 20.47 | 14.93 | 22.73 | 20.47 |

| **Pig Type** | **Drug** | **Pig** | **Dose**  **(mg)** | **T_max_**  **(hr)** | **C_max_**  **(ug/mL)** | **C_max_/D**  **(ug/mL/mg)** | **AUC_inf_**  **(day*ug/mL)** | **AUC_inf_/D**  **(day*ug/mL/mg)** | **T_1/2_**  **(day)** | **V_d_**  **(L)** | **CL**  **(L/day)** |
| --- | --- | --- | --- | --- | --- | --- | --- | --- | --- | --- | --- |
| Outbred | PB27 | 13 | 45.50 | 1.00 | 51.80 | 1.14 | 615.97 | 13.54 | 13.11 | 1.40 | 0.07 |
|  | | 14 | 49.00 | 0.03 | 50.16 | 1.02 | 671.04 | 13.69 | 14.41 | 1.52 | 0.07 |
|  |  | 15 | 45.50 | 0.03 | 45.15 | 0.99 | 753.91 | 16.57 | 19.67 | 1.71 | 0.06 |
|  |  | 16 | 42.00 | 0.03 | 48.20 | 1.15 | 889.07 | 21.17 | 16.36 | 1.11 | 0.05 |
|  |  | N | 4.00 | 4.00 | 4.00 | 4.00 | 4.00 | 4.00 | 4.00 | 4.00 | 4.00 |
|  |  | Min | 42.0 | 0.0333 | 45.1 | 0.992 | 616 | 13.5 | 13.1 | 1.11 | 0.0472 |
|  |  | Median | 45.5 | 0.0333 | 49.2 | 1.08 | 712 | 15.1 | 15.4 | 1.46 | 0.0667 |
|  |  | Max | 49.0 | 1.00 | 51.8 | 1.15 | 889 | 21.2 | 19.7 | 1.71 | 0.0739 |
|  |  | Geometric Mean | 45.43 | 0.07801 | 48.76 | 1.073 | 725.5 | 15.97 | 15.70 | 1.419 | 0.06262 |
|  |  | Geometric CV% | 6.302 | 412.7 | 5.929 | 7.392 | 15.98 | 21.18 | 17.67 | 18.26 | 21.18 |
